# Effects of the Forage Type and Chop Length of Ramie Silage on the Composition of Ruminal Microbiota in Black Goats

**DOI:** 10.3390/ani9040177

**Published:** 2019-04-18

**Authors:** Shengnan Sun, Zhenping Hou, Qiuzhong Dai, Duanqin Wu

**Affiliations:** 1 Institute of Bast Fiber Crops, Chinese Academy of Agricultural Sciences, Changsha 410205, China; snsun@yzu.edu.cn (S.S.); houzhenping@caas.cn (Z.H.); daiqiuzhong@caas.cn (Q.D.); 2 College of Animal Science and Technology, Yangzhou University, Yangzhou 225009, China

**Keywords:** bacterial community composition, black goats, chop length, ramie silage

## Abstract

**Simple Summary:**

The aim of this study was to evaluate the effects of the forage type and chop length of ramie (*Boehmeria nivea* (L.) Gaud.) silage on ruminal microbiota in black goats. The ruminal pH and relative abundance of microorganisms in the rumen differed with the lengths of the ramie silage fed to the goats. These results could provide a basis for ramie silage application in goats.

**Abstract:**

The aim of this study was to investigate the effects of the forage type and chop length of ramie (*Boehmeria nivea* (L.) Gaud.) silage on rumen fermentation and ruminal microbiota in black goats. Sixteen Liuyang black goats (22.35 ± 2.16 kg) were fed with the roughage of corn silage or ramie silage at chop lengths of 1, 2, or 3 cm. The Chao 1 index and the observed number of microbial species differed significantly between the corn and ramie silage groups (*p* < 0.05); however, *Firmicutes* (relative proportion: 34.99–56.68%), *Bacteroidetes* (27.41–47.73%), and *Proteobacteria* (1.44–3.92%) were the predominant phyla in both groups. The relative abundance of *Verrucomicrobia* (0.32–0.82%) was lowest for the 2 and 3 cm chop lengths (*p* < 0.05) and was negatively correlated with rumen pH and propionic acid concentration (*p* < 0.05), but positively correlated with the ratio of acetic acid to propionic acid (*p* < 0.05). The ramie silage fermentation quality was highest for the 1 cm chop length, suggesting that moderate chopping produces optimal quality silage.

## 1. Introduction

A large number of microorganisms, such as bacteria, fungi, and protozoa, inhabit the rumen, where they degrade the components of the plant cell wall, ensuring that the nutritional and energetic demands of ruminants are met [1]. Diet composition is one of the most important factors influencing the ruminal microbial community and its fermentation products. The forage source in particular has a considerable effect on the ruminal ecosystem [2,3]. In southern China (a subtropical zone), the climate is humid and hot, and is not suitable for the growth of high-quality grass such as alfalfa or Chinese wild rye; therefore, corn straw is often used as roughage to feed goats. However, corn straw is not only seasonal, but also of low nutritional value.

Ramie (*Boehmeria nivea* (L.) Gaud.) is a perennial herbaceous plant of the Urticaceae family and is native to China, Japan, and the Malay Peninsula where it has been used as a textile fiber for many centuries [4]. Ramie is also used as livestock forage because of the high crude protein content in its leaves and young stems [5]. Kipriotis reported that the nutritional value of ramie includes a 22% crude protein content, comprising stems and leaves on a dry matter basis, which exceeds that of alfalfa [6]. Ramie not only has a high nutritional value but can also be adapted to grow in southern China. The development of ramie as a forage resource is one way to alleviate the pressure of herbivores on the grasslands of southern China. Furthermore, silage production is a suitable method for ramie preservation and is used for ruminant husbandry, considering the climate of this region. However, few studies have evaluated how ramie silage could be efficiently used in animal production, especially regarding the different chop lengths of ramie silage. In the present study, the effects of two different forage types and the chop length of ramie silage on fermentation and microbial composition and structure in the rumen of black goats were investigated.

## 2. Materials and Methods

### 2.1. Animal Ethics

This study was carried out at the Institute of Bast Fiber Crops Animal Experimental Station (Yuanjiang city, Hunan Province, China). All experimental procedures and animal protocols were approved by the Animal Care and Use Committee (Permit No. 20170306), Institute of Bast Fiber Crops, Chinese Academy of Agricultural Sciences, Changsha, China.

### 2.2. Preparation of Silages

Ramie (Zhongsizhu No. 1) and maize were cultivated at the animal research station of the Institute of Bast Fiber Crops, Chinese Academy of Agricultural Sciences, Yuanjiang, Hunan Province, China (112°38’ E, 28°85’ N). Ramie was chopped into lengths of 1, 2, or 3 cm, and the silage was bagged for 60 days to prepare the total mixed ration (TMR). The ensiling method for the ramie was also used for maize with a chop length of 2 cm.

### 2.3. Animal and Experimental Design

Sixteen healthy Liuyang black goats (a local breed in Southern China) of a similar age with an average body weight of 22.35 ± 2.16 kg were chosen for the experiment. Four goats were randomly selected for each of the four treatments and were fed a roughage of corn silage (CON), or different chop lengths of ramie silage (R1, 1 cm; R2, 2 cm; and R3, 3 cm). Table 1 shows the composition and nutrient levels of the experimental diets. The goats were kept in individual metabolism pens (1.5 m × 2.0 m) with a diet trough and free access to fresh clean water throughout the experimental period. The feeding experiment was performed in two stages: a preliminary feeding period (14 days) and a formal experimental period (3 days for sampling).

### 2.4. Sampling and Measurements

Rumen fluid was collected through a polystyrene tube inserted into the rumen via the mouth after2 h of morning feeding. Approximately 50 mL of rumen content was obtained per animal. The pH of the rumen fluid was measured using a pH meter (Model S210 Seven Compact™ pH; Mettler-Toledo Instruments Ltd., Shanghai, China) immediately after sampling. The samples were then placed on dry ice and stored at −20 °C. Samples for volatile fatty acid (VFA) and ammonia nitrogen (NH_3_-N) analysis were prepared as described by Wu et al. [7], and 10 mL of the rumen contents was stored at −80 °C for DNA extraction.

### 2.5. DNA Extraction, Polymerase Chain Reaction (PCR), and Amplicon Sequencing

For each sample, total genomic DNA was extracted using a TIANamp stool DNA kit (TIANGEN, Beijing, China). The DNA concentration was determined using spectrophotometry (NanoDrop ND-1000, Nanodrop Technologies, Wilmington, DE, USA), and its quality was evaluated using 2% gel electrophoresis. Nuclease-free water was used as the blank for the spectrophotometric analysis. The total DNA was eluted in 50 µL of elution buffer and stored at −80 °C until measurement by PCR (LC-Bio Technology Co., Ltd., Hang Zhou, China).

### 2.6. PCR Amplication and 16s rDNA Sequencing

The V3–V4 region of the prokaryotic (bacterial and archaeal) small-subunit (16S) rRNA gene was amplified with slightly modified versions of the primers 338F (5′-ACTCCTACGGGAGGCAGCAG-3′) and 806R (5′-GGACTACHVGGGTWTCTAAT-3′) [8]. The 5′ ends of the primers were tagged with barcodes and sequencing universal primers specific to each sample. PCR amplification was performed in a total volume of 25 µL of reaction mixture containing 25 ng of template DNA, 12.5 µL of PCR Premix, 2.5 µL of each primer, and PCR-grade water to adjust the volume. The PCR conditions to amplify the prokaryotic 16S fragments consisted of an initial denaturation at 98 °C for 30 s, 35 cycles of denaturation at 98 °C for 10 s, annealing at 54 °C/52 °C for 30 s, and extension at 72 °C for 45 s, and then a final extension at 72 °C for 10 min. The presence of PCR products was confirmed by 2% agarose gel electrophoresis. Throughout the DNA extraction process, ultrapure water, instead of sample solution, was used as a negative control to exclude the possibility of false-positive PCR results. The PCR products were purified using AMPure XT beads (Beckman Coulter Genomics, Danvers, MA, USA) and quantified by Qubit (Invitrogen, Carisbad, CA, USA). The amplicon pools were prepared for sequencing, and the size and quantity of the amplicon library was assessed with an Agilent 2100 Bioanalyzer (Agilent, Santa Clara, CA, USA) and with the Library Quantification Kit for Illumina (Kapa Biosciences, Woburn, MA, USA), respectively. A PhiX Control library (v3) (Illumina) was combined with the amplicon library (expected at 30%). The libraries were sequenced on 300PE MiSeq runs and one library was sequenced with both protocols using the standard Illumina sequencing primers, eliminating the need for a third (or fourth) index read.

### 2.7. Sequence Analysis

The clean sequences were aligned into operational taxonomic units (OTUs) using the method of UCLUST based on 97% sequence similarity [9]. Alpha diversity indices [10] (Shannon–Wiener index, Chao 1 index, and the number of observed species) were calculated based on these sequences. The weighted UniFrac distance matrix between samples was calculated from the presence/absence of data and the abundance of OTUs, and visualized using Principal Coordinate Analysis (PCoA). All indices in our samples were calculated using QIIME open-source bioinformatics version 1.8.0.

### 2.8. Statistical Analysis

Data from rumen and fermenters were analyzed by one-way ANOVA with SPSS software (IBM SPSS Statistics v19; IBM Corp., Somers, NY, USA). Differences among means were considered significant at *p* < 0.05. When significant differences were detected in the one-way ANOVA, the differences among means were analyzed using the LSD comparison test and were considered significant at *p* < 0.1.

## 3. Results

### 3.1. Rumen Fermentation

**Figure 1:**
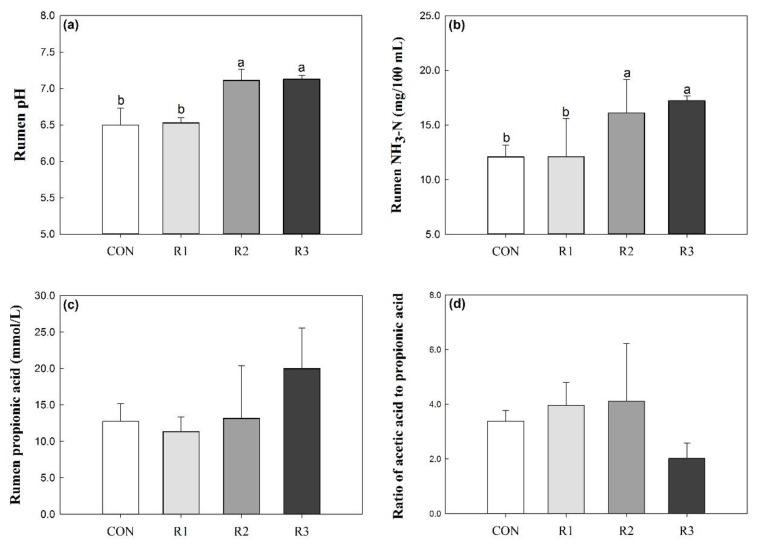
Effects of corn silage and different chop lengths of ramie silage on (**a**) rumen pH, (**b**) rumen NH_3_-N, (**c**) rumen propionic acid and (**d**) ratio of acetic acid to propionic acid. CON, corn silage; R1, 1 cm chopped ramie silage; R2, 2 cm chopped ramie silage; R3, 3 cm chopped ramie silage.

The rumen pH was not significantly different between CON and R1 groups (6.50 ± 0.12 for the CON group and 6.53 ± 0.04 for the R1 group). However, the rumen pH in the R2 and R3 groups was 7.11 ± 0.08 and 7.13 ± 0.03, respectively, and these values were significantly different from those of the CON and R1 groups (Figure 1a, *p* < 0.05). The concentration of rumen NH_3_–N was significantly higher in the R2 and R3 groups than in the CON and R1 groups (Figure 1b, *p* < 0.05). The concentration of propionic acid and the ratio of acetic acid to propionic acid in the rumen of the goats were not influenced by the different diets (Figure 1c,d, *p* > 0.05).

### 3.2. Alpha Diversity of Ruminal Microbiota

The Shannon–Wiener index, used to indicate the species-richness and -evenness of the samples, tended to be lower (*p* = 0.10) in the CON group than in the other groups (Table 2). The Chao 1 index and the number of observed species were influenced by the different diets, being lower (*p* < 0.05) in the CON group than in the other groups (Table 2).

### 3.3. Rumen Microbial Composition at the Phylum Level

**Figure 2:**
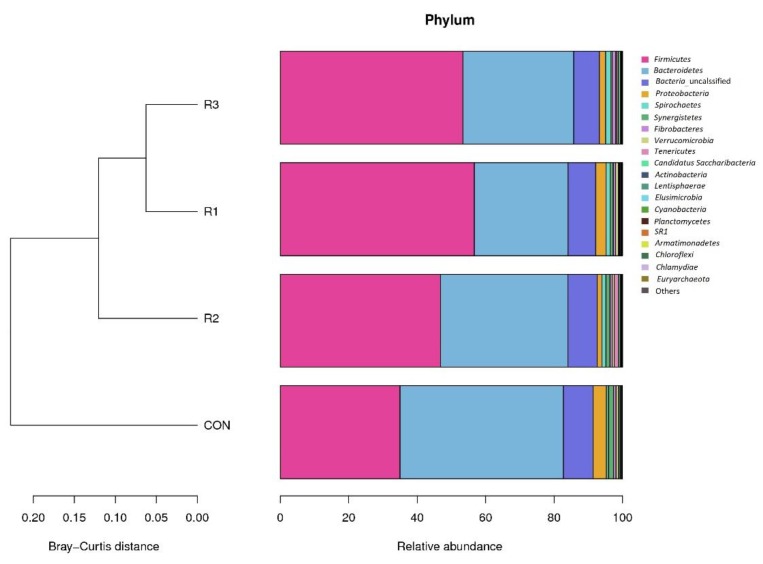
The relative abundance at the phylum level of rumen microbial communities in goats fed with corn and ramie. CON, corn silage; R1, 1 cm chopped ramie silage; R2, 2 cm chopped ramie silage; R3, 3 cm chopped ramie silage.

The composition and relative abundance of microorganisms at the phylum level are presented in Table 3 and Figure 2. *Firmicutes* was the predominant phylum in the R1, R2, and R3 groups and tended (*p* = 0.07) to have greater abundance in these groups than in the CON group (Table 3, Figure 2). *Bacteroidetes* was the predominant phylum in the CON group, which tended (*p* = 0.06) to have a greater abundance in this group than in the R1, R2, and R3 groups (Table 3, Figure 2). 

The third most abundant phylum was *Proteobacteria*, followed by *Spirochaetes*, *Synergistetes*, and *Verrucomicrobia* in decreasing order of abundance. Only two phyla exhibited significantly different relative abundances among the CON, R1, R2, and R3 groups. The relative abundance of *Lentisphaerae* was higher in the CON group than in the R3 group (*p* < 0.001), and that of *Verrucomicrobia* was lower in the R2 and R3 groups than in the CON and R1 groups (*p* = 0.04). In the Kingdom Archaea, only one phylum, *Euryarchaeota*, was detected in the R1, R2, and R3 groups, and its relative abundance was not influenced by the different diets (*p* = 0.36).

### 3.4. Effects of Silage Type and Chop Length on Verrucomicrobia

**Figure 3:**
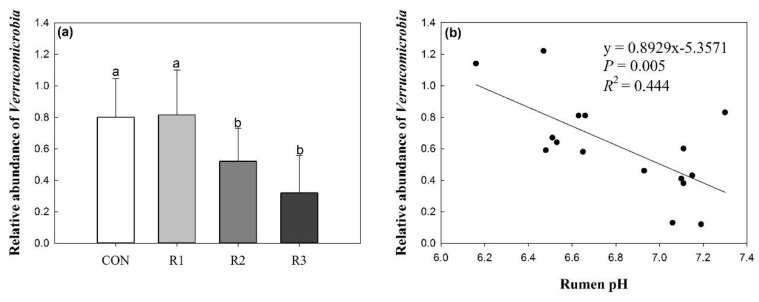

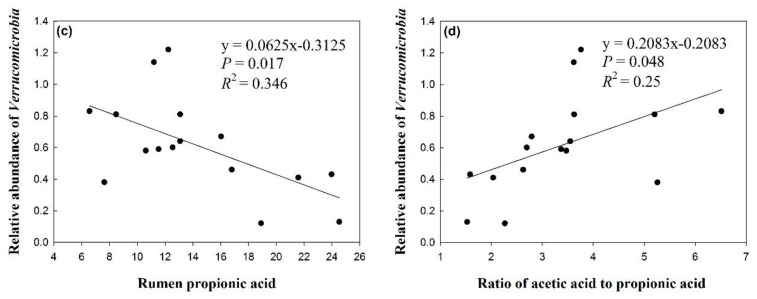
Effects of silage type and chop length on *Verrucomicrobia* (**a**), and the relationships between the relative abundance of *Verrucomicrobia* and rumen fermentation (**b**, **c**, and **d**). CON, corn silage; R1, 1 cm chopped ramie silage; R2, 2 cm chopped ramie silage; R3, 3 cm chopped ramie silage.

The relative abundance of *Verrucomicrobia* was significantly lower in ramie silage with 2 and 3 cm chop lengths (Figure 3a, *p* < 0.05)**,** and was significantly negatively correlated with the rumen pH and propionic acid concentration (Figure 3b,c, *p* < 0.05), but positively correlated with the ratio of acetic acid to propionic acid in the rumen (Figure 3d, *p* < 0.05).

### 3.5. Analysis of Weighted UniFrac Distance

**Figure 4:**
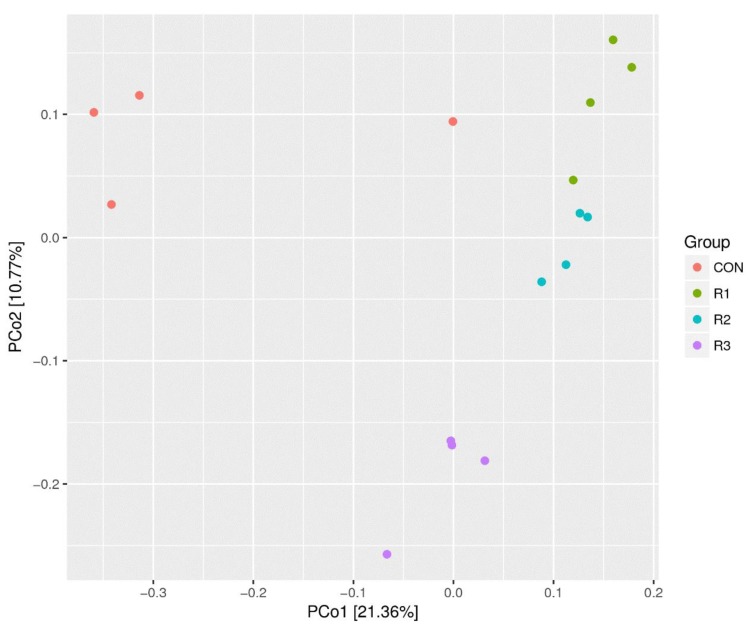
Principal coordinate analysis plot of total microbiota in rumen samples of goats fed with corn and different chop lengths of ramie. CON, corn silage; R1, 1 cm chopped ramie silage; R2, 2 cm chopped ramie silage; R3, 3 cm chopped ramie silage.

The analysis of rumen microbiota beta diversity was visualized using a PCoA plot (Figure 4). The first component separated samples from the CON and R1, R2, and R3 groups and explained 21.36% of the total variation. The second component, which explained 10.77% of the total variation, separated the individuals within the R3 group from each other.

## 4. Discussion

In our previous study, we found that the length of feeding ramie silage had no significant effect on the average daily intake of goats (*p* > 0.05) [11]. The pH of rumen fluid is the most basic index reflecting the rumen fermentation status of ruminants, which is indicated by the formation and absorption of organic acids (such as VFAs) in the rumen, the amount of saliva, and the amount of rumen contents from the rumen to the hindgut [12]. A previous study showed that corn stover decreased the ruminal pH to a greater extent than alfalfa hay in Hu sheep [13]. The dynamic variation in rumen pH was also observed in the present study. This variation may be because corn stover is rich in sugar and is easily fermented to produce organic acids. Our findings are similar to those of Soita et al. [14], who fed different lengths of barley or corn silage to cattle. Soita et al. [14] and Schwab et al. [15] found that the ruminal pH increased in cattle with increasing lengths of silage forage.

In a study conducted by Einarson et al. [16], barley silage was used as forage and was fed to lactating dairy cows at chop lengths of 2 cm. The reduction in chop length decreased the ruminal NH_3_–N concentration. In addition, Thomson et al. [17] reported that when lactating dairy cows were fed TMRs comprising lucerne and maize silage DM in proportions (wt/wt) of 25:75, the short chop length of lucerne silage decreased the concentration of rumen NH_3_ relative to the long chop length. This indicated that increasing the length of the roughage extended the fermentation time of forage in the rumen, improved protein digestibility, and increased the ruminal NH_3_–N concentration. Krause et al. [18] found that increasing the dietary particle size decreased the total ruminal VFA and increased the acetate-to-propionate ratio in midlactation cows. In contrast, Beauchemin et al. [19] confirmed that differing alfalfa particle sizes had no effect on the total VFA and acetate-to-propionate ratio in dairy cows. These contrasting results might be explained by the many factors affecting ruminal VFA, such as forage source, animal species, and rumen volume, and flow rate. 

Variations in the composition of rumen bacteria depending on the diet provided to the animal have been reported previously [20]. Jin et al. [21] found that rumen bacteria differed significantly in cows fed with corn stover from that in those fed with mixed forage (alfalfa hay and corn silage). Abecia et al. [22] investigated the effects of forage type on diversity in bacterial pellets isolated from the rumen contents of sheep and goats and confirmed that feeding the animals alfalfa hay tended (*p* < 0.10) to promote a higher Shannon index or diversity in the pellets than when the animals were fed with grass hay. In the present study, the ramie silage groups revealed a higher observed bacterial species diversity than the corn silage group. A similar study also showed that rumen bacterial diversity was higher in sheep fed with alfalfa hay than those fed with grass hay [23]. The ruminal bacteria that attach to feed particles aid in the digestion of plant cell walls. Thus, the chemical composition and anatomical structure of different forages affect ruminal digestion. The higher quality forage is easier to digest in the rumen, while at the same time, it is more beneficial to microbial reproduction.

In the present study, *Firmicutes* and *Bacteroidetes* were the two dominant phyla and together accounted for 90% of the microorganisms in the rumen. These findings are in accordance with the findings of previous studies, indicating that they are the most important phyla in the rumen [24,25]. Kong et al. [26] also identified *Firmicutes* and *Bacteroidetes* as the two predominant bacteria phyla in the rumen fraction of cows fed with alfalfa or triticale. *Firmicutes* includes numerous fiber-adherent bacterial species involved in the degradation of various polysaccharides which are commonly found in ruminants fed with forage diets [24,27]. Grilli et al. [28] investigated goats fed diets with fiber-to-concentrate (grain) ratios of 100:0 (AH diet) or 60:40 (AH/C diet) and found that *Verrucomicrobia* was only detected in the AH/C diet-fed animals. In the present study, similar results were obtained from the rumen contents of animals fed diets with forage-to-concentrate (grain) ratios of 60:40. Furthermore, the relative abundance of *Verrucomicrobia* was negatively correlated with the pH and concentration of propionic acid in the rumen, but positively correlated with the ratio of propionic acid to acetic acid (Figure 3b–d). Overall, our results showed that rumen bacterial diversity was affected by the chop length to some extent; however, the main structure and composition of rumen microbes in the goats remained stable across the different treatments. The PCoA analysis also showed that the distance between CON and ramie groups was much larger than the distance between the different chop length groups (Figure 4). This indicates that the forage type does affect the rumen bacterial diversity, which is in accordance with other studies [29,30].

## 5. Conclusions

Our results showed that the forage type had some effects on the rumen pH and NH_3_–N concentration, especially for corn silage relative to ramie silage with 2 and 3 cm chop lengths. The rumen bacterial profile and diversity were influenced by the different types of forage in the diet. *Firmicutes* abundance tended to increase in goats fed with ramie silage when compared to those fed with corn silage, while *Bacteroidetes* showed the opposite tendency.

## Figures and Tables

**Table 1:** Composition and nutrient levels of the experimental diet *.

Items	CON	R1	R2	R3
Ingredient (%)				
Corn	20.4	20.4	20.4	20.4
Wheat bran	10.72	12.72	12.72	12.72
Soybean meal	6.8	4.8	4.8	4.8
NaCl	0.48	0.48	0.48	0.48
Premix	1.6	1.6	1.6	1.6
Ramie silage		60.0	60.0	60.0
Corn silage	60.0			
Total	100	100.0	100.0	100.0
Nutrient levels				
ME (MJ/Kg)	8.71	8.72	8.72	8.72
CP (%)	15.0	14.9	15.2	14.9
Starch (%)	21.2	20.7	20.7	20.7
NDF (%)	37.0	37.5	36.7	37.3
ADF (%)	26.1	26.6	26.4	26.8
Ca (%)	2.85	2.87	2.86	2.88
P (%)	0.40	0.31	0.32	0.31

* CON, corn silage; R1, 1 cm chopped ramie silage; R2, 2 cm chopped ramie silage; R3, 3 cm chopped ramie silage. The premix contained the following per kg: 120 g of MgSO_4_·H_2_O, 3 g of FeSO_4_·7H_2_O, 1 g of CuSO_4_·5H_2_O, 3 g of MnSO_4_·H_2_O, 5 g of ZnSO_4_·H_2_O, 10 mg of Na_2_SeO_3_, 40 mg of KI, 30 mg of CoCl_2_·6H_2_O, 100,000 IU vitamin A, 18,000 IU vitamin D, 20,000 IU vitamin E.

**Table 2:** Alpha diversity indices of rumen microbial communities in goats fed with corn and ramie, calculated from 7229 sequences (*n* = 4) *.

	CON	R1	R2	R3	SEM	*p*-Value
Shannon-Wiener	8.73	9.25	9.26	9.18	0.36	0.10
Chao 1	2632.79	3649.97	3956.94	3398.12	634.69	<0.01
Observed Species	2050.00	2730.50	2826.00	2547.25	412.63	0.02

* CON, corn silage; R1, 1 cm chopped ramie silage; R2, 2 cm chopped ramie silage; R3, 3 cm chopped ramie silage; SEM, standard error of the means.

**Table 3:** Composition and relative abundance at the phylum level of the rumen microbial communities in goats fed with corn and ramie (*n* = 4) *.

Kingdom	Phylum	CON	R1	R2	R3	SEM	*p*-Value
Bacteria	*Firmicutes*	34.99	56.68	46.85	53.33	13.06	0.07
	*Bacteroidetes*	47.73	27.41	37.16	32.39	11.50	0.06
	*Proteobacteria*	3.92	3.02	1.44	1.84	1.81	0.20
	*Spirochaetes*	0.62	1.23	1.15	1.49	0.68	0.35
	*Synergistetes*	1.42	0.90	1.18	0.45	0.72	0.27
	*Fibrobacteres*	0.78	0.71	0.69	1.02	0.70	0.92
	*Verrucomicrobia*	0.80	0.82	0.52	0.32	0.31	0.04
	*Tenericutes*	0.06	0.24	1.37	0.46	0.91	0.17
	*Candidatus Saccharibacteria*	0.38	0.19	0.47	0.56	0.42	0.69
	*Actinobacteria*	0.14	0.32	0.14	0.22	0.12	0.10
	*Lentisphaerae*	0.29	0.13	0.12	0.06	0.10	<0.001
	*Elusimicrobia*	0.10	0.13	0.18	0.06	0.12	0.57
	*Cyanobacteria*	0.09	0.08	0.13	0.16	0.07	0.47
	*Planctomycetes*	0.01	0.04	0.01	0.05	0.04	0.34
	*SR1*	0.00	0.01	0.02	0.05	0.05	0.56
	*Armatimonadetes*	0.01	0.02	0.01	0.02	0.01	0.82
	*Chloroflexi*	0.01	0.01	0.01	0.01	0.01	0.75
	*Chlamydiae*	0.00	0.02	0.005	0.00	0.20	0.40
Archaea	*Euryarchaeota*	0.00	0.01	0.005	0.01	0.01	0.36

* CON, corn silage; R1, 1 cm chopped ramie silage; R2, 2 cm chopped ramie silage; R3, 3 cm chopped ramie silage; SEM, standard error of the means.
